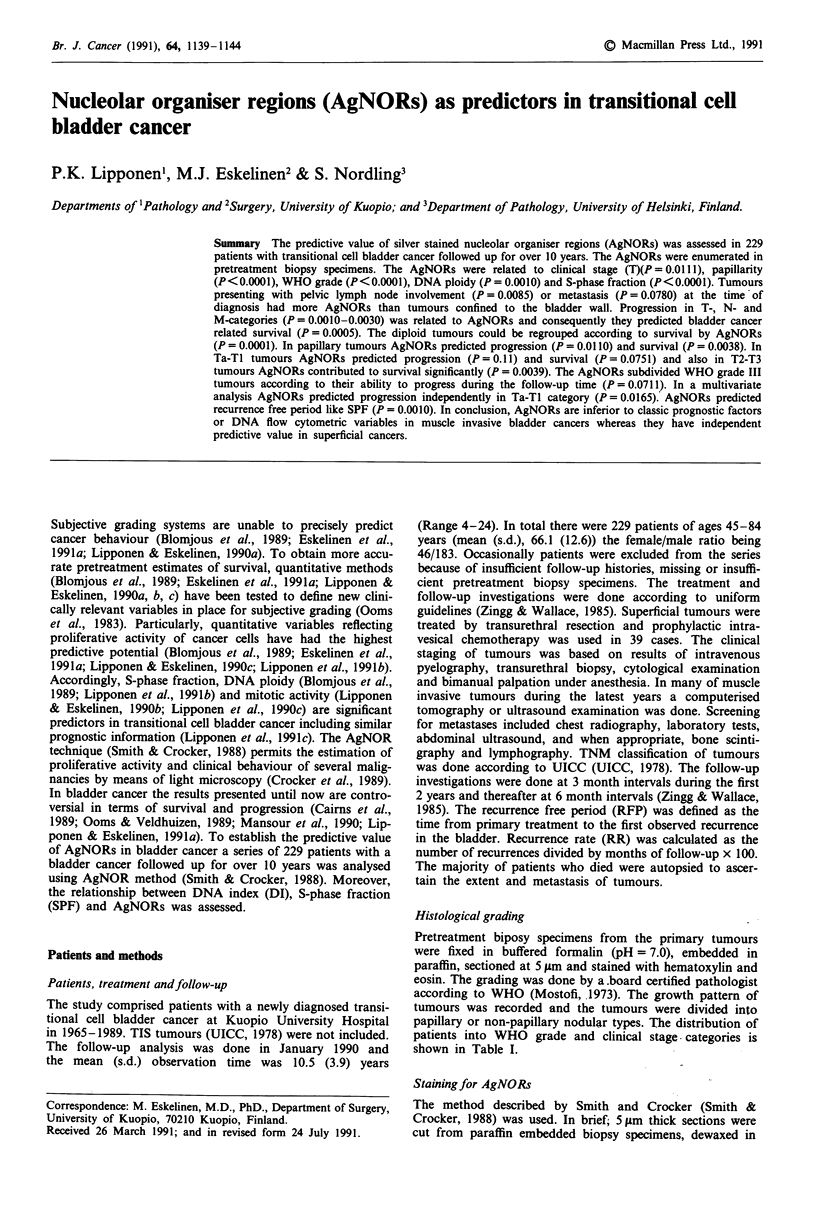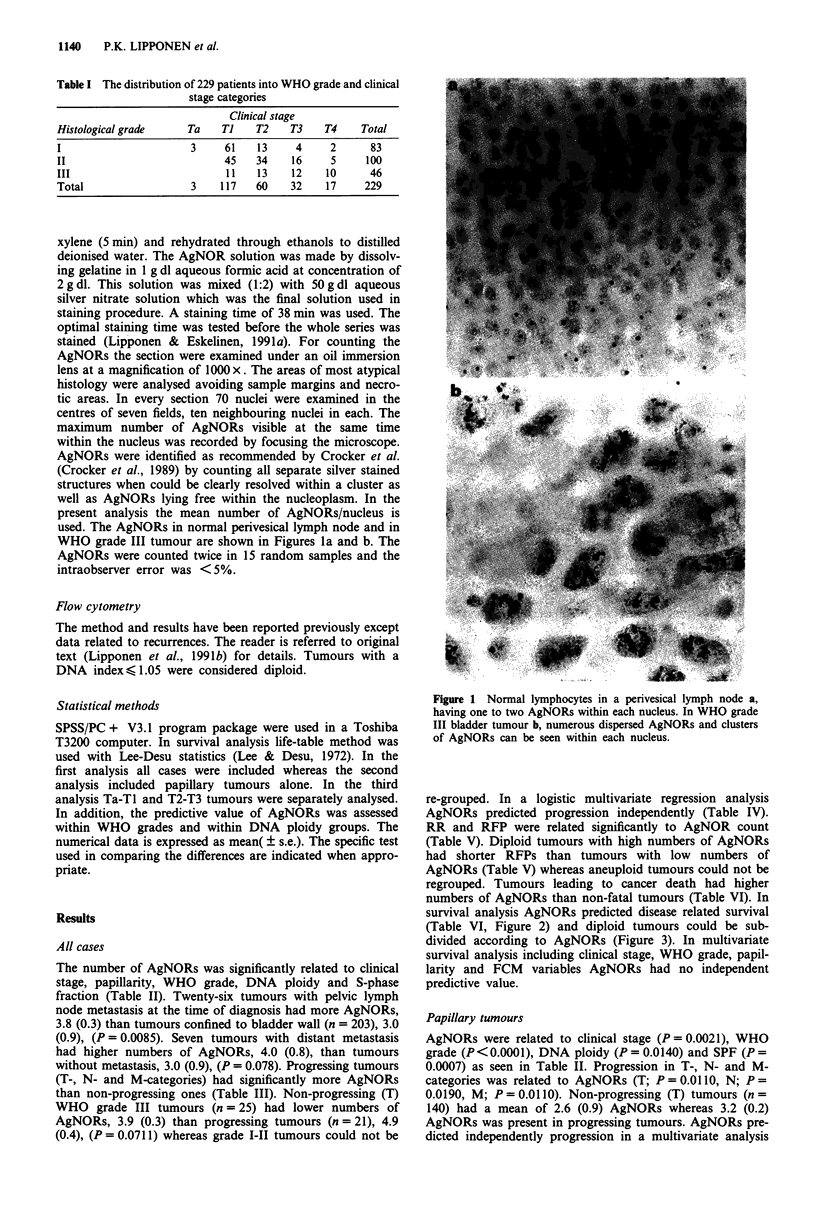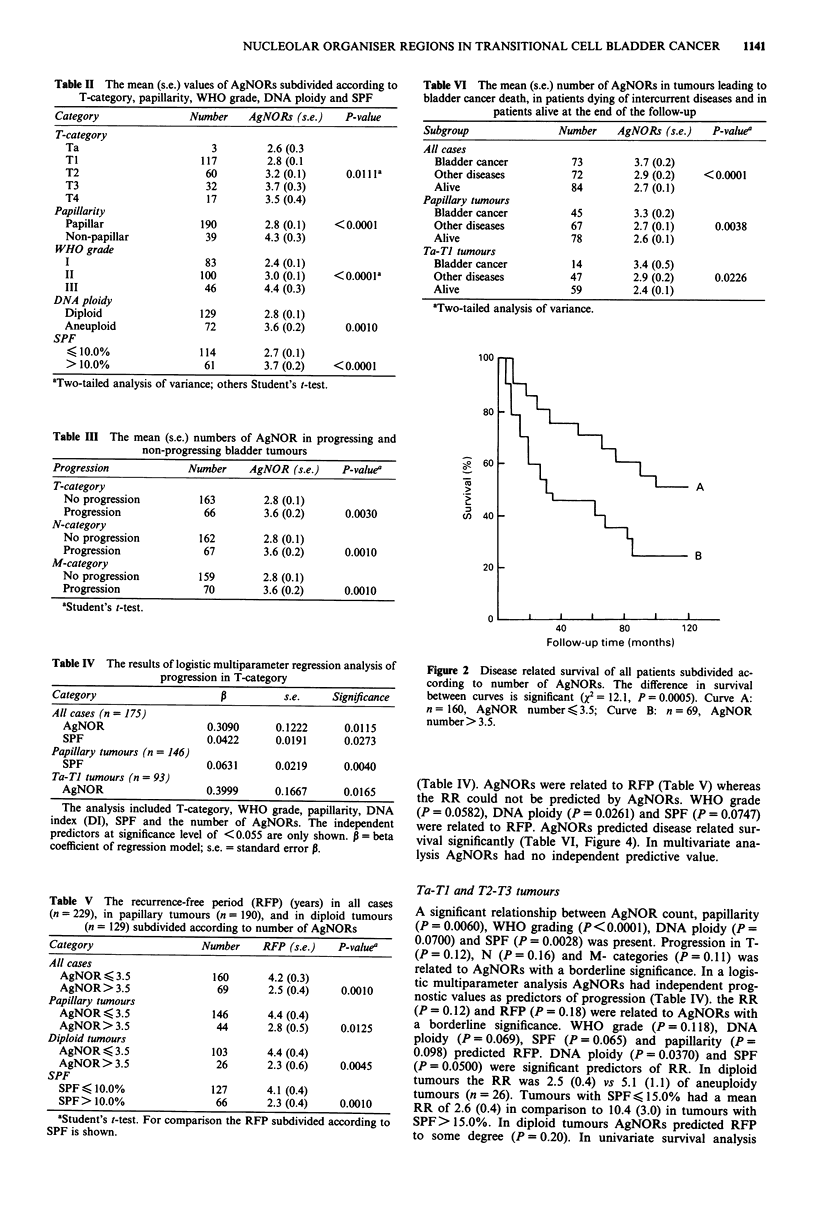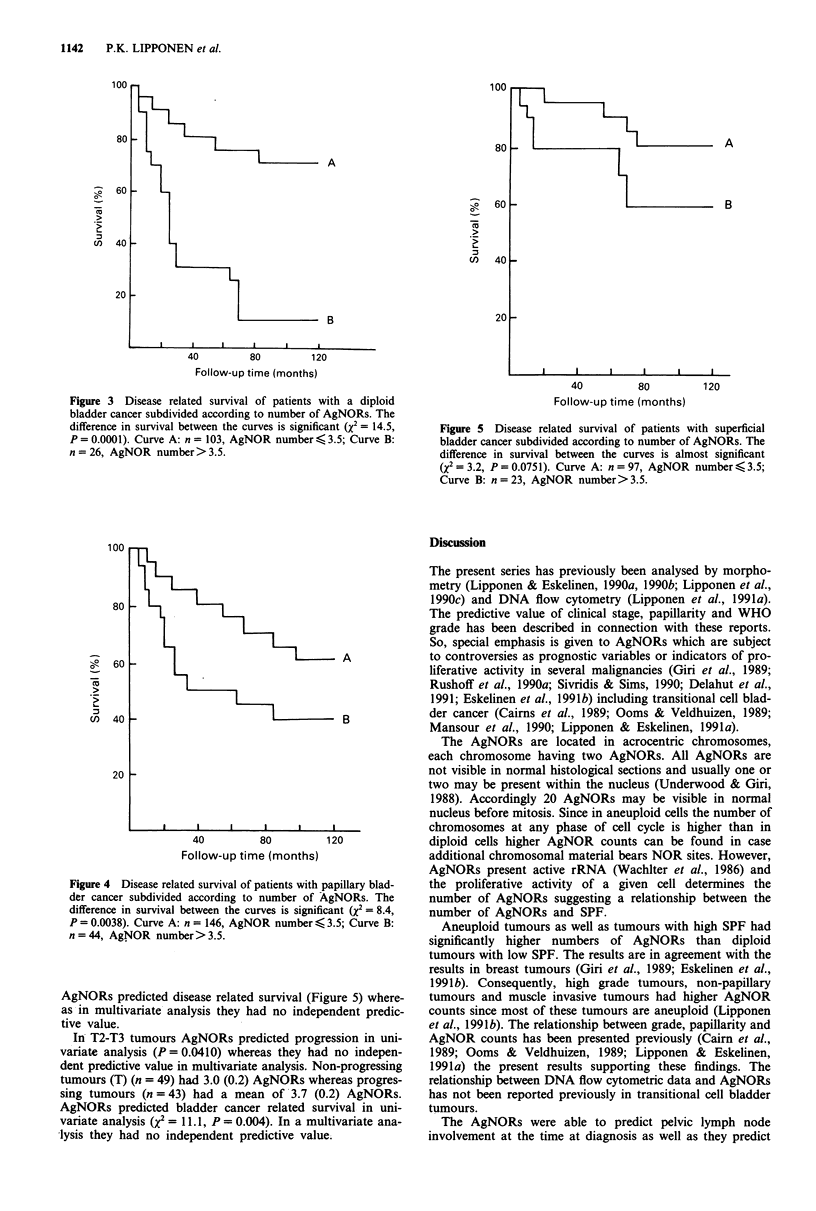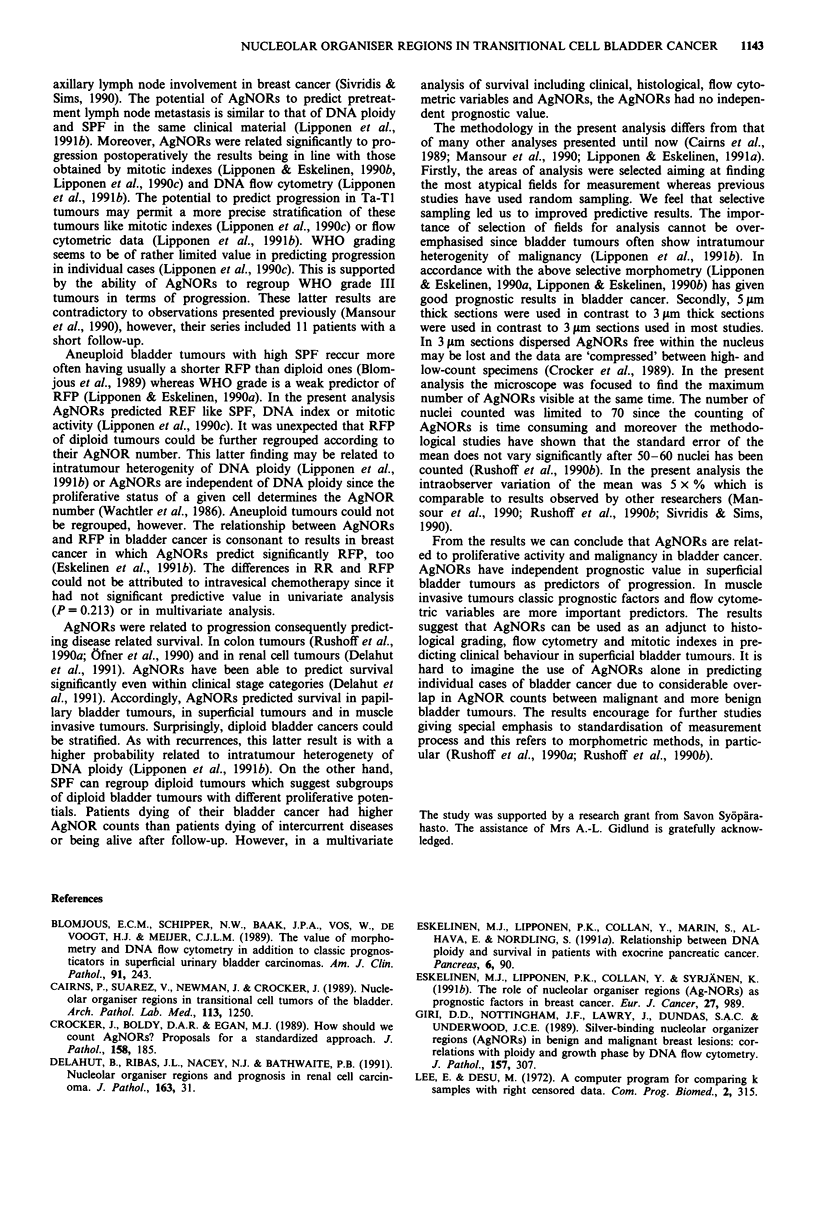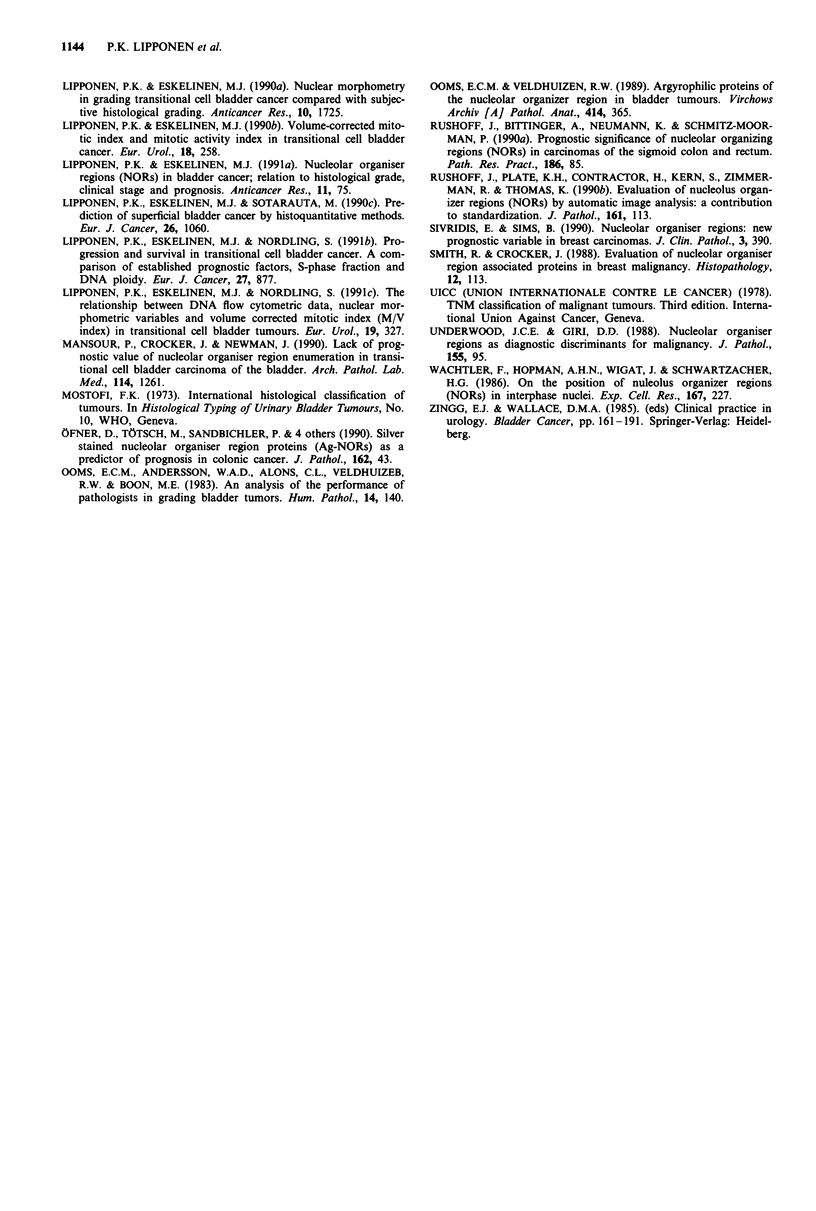# Nucleolar organiser regions (AgNORs) as predictors in transitional cell bladder cancer.

**DOI:** 10.1038/bjc.1991.478

**Published:** 1991-12

**Authors:** P. K. Lipponen, M. J. Eskelinen, S. Nordling

**Affiliations:** Department of Pathology, University of Kuopio, Finland.

## Abstract

**Images:**


*Br. J. Cancer* (1991), **64**, 1139–1144 © Macmillan Press Ltd., 1991

### Nucleolar organiser regions (AgNORs) as predictors in transitional cell bladder cancer

P.K. Lipponen[1], M.J. Eskelinen[2] & S. Nordling[3]

*Departments of [1]Pathology and [2]Surgery, University of Kuopio; and [3]Department of Pathology, University of Helsinki, Finland.*

**Summary** The predictive value of silver stained nucleolar organiser regions (AgNORs) was assessed in 229 patients with transitional cell bladder cancer followed up for over 10 years. The AgNORs were enumerated in pretreatment biopsy specimens. The AgNORs were related to clinical stage (T)($P = 0.0111$), papillarity ($P < 0.0001$), WHO grade ($P < 0.0001$), DNA ploidy ($P = 0.0010$) and S-phase fraction ($P < 0.0001$). Tumours presenting with pelvic lymph node involvement ($P = 0.0085$) or metastasis ($P = 0.0780$) at the time of diagnosis had more AgNORs than tumours confined to the bladder wall. Progression in T-, N- and M-categories ($P = 0.0010–0.0030$) was related to AgNORs and consequently they predicted bladder cancer related survival ($P = 0.0005$). The diploid tumours could be regrouped according to survival by AgNORs ($P = 0.0001$). In papillary tumours AgNORs predicted progression ($P = 0.0110$) and survival ($P = 0.0038$). In Ta-T1 tumours AgNORs predicted progression ($P = 0.11$) and survival ($P = 0.0751$) and also in T2-T3 tumours AgNORs contributed to survival significantly ($P = 0.0039$). The AgNORs subdivided WHO grade III tumours according to their ability to progress during the follow-up time ($P = 0.0711$). In a multivariate analysis AgNORs predicted progression independently in Ta-T1 category ($P = 0.0165$). AgNORs predicted recurrence free period like SPF ($P = 0.0010$). In conclusion, AgNORs are inferior to classic prognostic factors or DNA flow cytometric variables in muscle invasive bladder cancers whereas they have independent predictive value in superficial cancers.

Subjective grading systems are unable to precisely predict cancer behaviour (Blomjous *et al.*, 1989; Eskelinen *et al.*, 1991*a*; Lipponen & Eskelinen, 1990*a*). To obtain more accurate pretreatment estimates of survival, quantitative methods (Blomjous *et al.*, 1989; Eskelinen *et al.*, 1991*a*; Lipponen & Eskelinen, 1990*a*, *b*, *c*) have been tested to define new clinically relevant variables in place for subjective grading (Ooms *et al.*, 1983). Particularly, quantitative variables reflecting proliferative activity of cancer cells have had the highest predictive potential (Blomjous *et al.*, 1989; Eskelinen *et al.*, 1991*a*; Lipponen & Eskelinen, 1990*c*; Lipponen *et al.*, 1991*b*). Accordingly, S-phase fraction, DNA ploidy (Blomjous *et al.*, 1989; Lipponen *et al.*, 1991*b*) and mitotic activity (Lipponen & Eskelinen, 1990*b*; Lipponen *et al.*, 1990*c*) are significant predictors in transitional cell bladder cancer including similar prognostic information (Lipponen *et al.*, 1991*c*). The AgNOR technique (Smith & Crocker, 1988) permits the estimation of proliferative activity and clinical behaviour of several malignancies by means of light microscopy (Crocker *et al.*, 1989). In bladder cancer the results presented until now are controversial in terms of survival and progression (Cairns *et al.*, 1989; Ooms & Veldhuizen, 1989; Mansour *et al.*, 1990; Lipponen & Eskelinen, 1991*a*). To establish the predictive value of AgNORs in bladder cancer a series of 229 patients with a bladder cancer followed up for over 10 years was analysed using AgNOR method (Smith & Crocker, 1988). Moreover, the relationship between DNA index (DI), S-phase fraction (SPF) and AgNORs was assessed.

### Patients and methods

### *Patients, treatment and follow-up*

The study comprised patients with a newly diagnosed transitional cell bladder cancer at Kuopio University Hospital in 1965–1989. TIS tumours (UICC, 1978) were not included. The follow-up analysis was done in January 1990 and the mean (s.d.) observation time was 10.5 (3.9) years (Range 4–24). In total there were 229 patients of ages 45–84 years (mean (s.d.), 66.1 (12.6)) the female/male ratio being 46/183. Occasionally patients were excluded from the series because of insufficient follow-up histories, missing or insufficient pretreatment biopsy specimens. The treatment and follow-up investigations were done according to uniform guidelines (Zingg & Wallace, 1985). Superficial tumours were treated by transurethral resection and prophylactic intravesical chemotherapy was used in 39 cases. The clinical staging of tumours was based on results of intravenous pyelography, transurethral biopsy, cytological examination and bimanual palpation under anesthesia. In many of muscle invasive tumours during the latest years a computerised tomography or ultrasound examination was done. Screening for metastases included chest radiography, laboratory tests, abdominal ultrasound, and when appropriate, bone scintigraphy and lymphography. TNM classification of tumours was done according to UICC (UICC, 1978). The follow-up investigations were done at 3 month intervals during the first 2 years and thereafter at 6 month intervals (Zingg & Wallace, 1985). The recurrence free period (RFP) was defined as the time from primary treatment to the first observed recurrence in the bladder. Recurrence rate (RR) was calculated as the number of recurrences divided by months of follow-up × 100. The majority of patients who died were autopsied to ascertain the extent and metastasis of tumours.

### *Histological grading*

Pretreatment bipsoy specimens from the primary tumours were fixed in buffered formalin (pH = 7.0), embedded in paraffin, sectioned at 5 μm and stained with hematoxylin and eosin. The grading was done by a board certified pathologist according to WHO (Mostofi, 1973). The growth pattern of tumours was recorded and the tumours were divided into papillary or non-papillary nodular types. The distribution of patients into WHO grade and clinical stage categories is shown in Table I.

### *Staining for AgNORs*

The method described by Smith and Crocker (Smith & Crocker, 1988) was used. In brief; 5 μm thick sections were cut from paraffin embedded biopsy specimens, dewaxed in

Correspondence: M. Eskelinen, M.D., PhD., Department of Surgery, University of Kuopio, 70210 Kuopio, Finland.
Received 26 March 1991; and in revised form 24 July 1991.

**Table I** The distribution of 229 patients into WHO grade and clinical stage categories

| | *Clinical stage* | | | | | |
|---|---|---|---|---|---|---|
| *Histological grade* | *Ta* | *T1* | *T2* | *T3* | *T4* | *Total* |
| I | 3 | 61 | 13 | 4 | 2 | 83 |
| II | | 45 | 34 | 16 | 5 | 100 |
| III | | 11 | 13 | 12 | 10 | 46 |
| Total | 3 | 117 | 60 | 32 | 17 | 229 |

xylene (5 min) and rehydrated through ethanols to distilled deionised water. The AgNOR solution was made by dissolving gelatine in 1 g dl aqueous formic acid at concentration of 2 g dl. This solution was mixed (1:2) with 50 g dl aqueous silver nitrate solution which was the final solution used in staining procedure. A staining time of 38 min was used. The optimal staining time was tested before the whole series was stained (Lipponen & Eskelinen, 1991*a*). For counting the AgNORs the section were examined under an oil immersion lens at a magnification of 1000 ×. The areas of most atypical histology were analysed avoiding sample margins and necrotic areas. In every section 70 nuclei were examined in the centres of seven fields, ten neighbouring nuclei in each. The maximum number of AgNORs visible at the same time within the nucleus was recorded by focusing the microscope. AgNORs were identified as recommended by Crocker *et al.* (Crocker *et al.*, 1989) by counting all separate silver stained structures when could be clearly resolved within a cluster as well as AgNORs lying free within the nucleoplasm. In the present analysis the mean number of AgNORs/nucleus is used. The AgNORs in normal perivesical lymph node and in WHO grade III tumour are shown in Figures 1a and b. The AgNORs were counted twice in 15 random samples and the intraobserver error was $<5\%$.

### *Flow cytometry*

The method and results have been reported previously except data related to recurrences. The reader is referred to original text (Lipponen *et al.*, 1991*b*) for details. Tumours with a DNA index $\leqslant 1.05$ were considered diploid.

### *Statistical methods*

SPSS/PC + V3.1 program package were used in a Toshiba T3200 computer. In survival analysis life-table method was used with Lee-Desu statistics (Lee & Desu, 1972). In the first analysis all cases were included whereas the second analysis included papillary tumours alone. In the third analysis Ta-T1 and T2-T3 tumours were separately analysed. In addition, the predictive value of AgNORs was assessed within WHO grades and within DNA ploidy groups. The numerical data is expressed as mean( ± s.e.). The specific test used in comparing the differences are indicated when appropriate.

### Results

### *All cases*

The number of AgNORs was significantly related to clinical stage, papillarity, WHO grade, DNA ploidy and S-phase fraction (Table II). Twenty-six tumours with pelvic lymph node metastasis at the time of diagnosis had more AgNORs, 3.8 (0.3) than tumours confined to bladder wall ($n = 203$), 3.0 (0.9), ($P = 0.0085$). Seven tumours with distant metastasis had higher numbers of AgNORs, 4.0 (0.8), than tumours without metastasis, 3.0 (0.9), ($P = 0.078$). Progressing tumours (T-, N- and M-categories) had significantly more AgNORs than non-progressing ones (Table III). Non-progressing (T) WHO grade III tumours ($n = 25$) had lower numbers of AgNORs, 3.9 (0.3) than progressing tumours ($n = 21$), 4.9 (0.4), ($P = 0.0711$) whereas grade I-II tumours could not be

**Figure 1** Normal lymphocytes in a perivesical lymph node **a**, having one to two AgNORs within each nucleus. In WHO grade III bladder tumour **b**, numerous dispersed AgNORs and clusters of AgNORs can be seen within each nucleus.

re-grouped. In a logistic multivariate regression analysis AgNORs predicted progression independently (Table IV). RR and RFP were related significantly to AgNOR count (Table V). Diploid tumours with high numbers of AgNORs had shorter RFPs than tumours with low numbers of AgNORs (Table V) whereas aneuploid tumours could not be regrouped. Tumours leading to cancer death had higher numbers of AgNORs than non-fatal tumours (Table VI). In survival analysis AgNORs predicted disease related survival (Table VI, Figure 2) and diploid tumours could be subdivided according to AgNORs (Figure 3). In multivariate survival analysis including clinical stage, WHO grade, papillarity and FCM variables AgNORs had no independent predictive value.

### *Papillary tumours*

AgNORs were related to clinical stage ($P = 0.0021$), WHO grade ($P<0.0001$), DNA ploidy ($P = 0.0140$) and SPF ($P = 0.0007$) as seen in Table II. Progression in T-, N- and M-categories was related to AgNORs (T; $P = 0.0110$, N; $P = 0.0190$, M; $P = 0.0110$). Non-progressing (T) tumours ($n = 140$) had a mean of 2.6 (0.9) AgNORs whereas 3.2 (0.2) AgNORs was present in progressing tumours. AgNORs predicted independently progression in a multivariate analysis

**Table II** The mean (s.e.) values of AgNORs subdivided according to T-category, papillarity, WHO grade, DNA ploidy and SPF

| *Category* | *Number* | *AgNORs (s.e.)* | *P-value* |
|---|---|---|---|
| *T-category* | | | |
| Ta | 3 | 2.6 (0.3 | |
| T1 | 117 | 2.8 (0.1 | |
| T2 | 60 | 3.2 (0.1) | 0.0111[a] |
| T3 | 32 | 3.7 (0.3) | |
| T4 | 17 | 3.5 (0.4) | |
| *Papillarity* | | | |
| Papillar | 190 | 2.8 (0.1) | <0.0001 |
| Non-papillar | 39 | 4.3 (0.3) | |
| *WHO grade* | | | |
| I | 83 | 2.4 (0.1) | |
| II | 100 | 3.0 (0.1) | <0.0001[a] |
| III | 46 | 4.4 (0.3) | |
| *DNA ploidy* | | | |
| Diploid | 129 | 2.8 (0.1) | |
| Aneuploid | 72 | 3.6 (0.2) | 0.0010 |
| *SPF* | | | |
| ≤10.0% | 114 | 2.7 (0.1) | |
| >10.0% | 61 | 3.7 (0.2) | <0.0001 |

[a]Two-tailed analysis of variance; others Student's *t*-test.

**Table III** The mean (s.e.) numbers of AgNOR in progressing and non-progressing bladder tumours

| *Progression* | *Number* | *AgNOR (s.e.)* | *P-value*[a] |
|---|---|---|---|
| *T-category* | | | |
| No progression | 163 | 2.8 (0.1) | |
| Progression | 66 | 3.6 (0.2) | 0.0030 |
| *N-category* | | | |
| No progression | 162 | 2.8 (0.1) | |
| Progression | 67 | 3.6 (0.2) | 0.0010 |
| *M-category* | | | |
| No progression | 159 | 2.8 (0.1) | |
| Progression | 70 | 3.6 (0.2) | 0.0010 |

[a]Student's *t*-test.

**Table IV** The results of logistic multiparameter regression analysis of progression in T-category

| *Category* | *β* | *s.e.* | *Significance* |
|---|---|---|---|
| *All cases (n = 175)* | | | |
| AgNOR | 0.3090 | 0.1222 | 0.0115 |
| SPF | 0.0422 | 0.0191 | 0.0273 |
| *Papillary tumours (n = 146)* | | | |
| SPF | 0.0631 | 0.0219 | 0.0040 |
| *Ta-T1 tumours (n = 93)* | | | |
| AgNOR | 0.3999 | 0.1667 | 0.0165 |

The analysis included T-category, WHO grade, papillarity, DNA index (DI), SPF and the number of AgNORs. The independent predictors at significance level of <0.055 are only shown. $\beta$ = beta coefficient of regression model; s.e. = standard error $\beta$.

**Table V** The recurrence-free period (RFP) (years) in all cases ($n$ = 229), in papillary tumours ($n$ = 190), and in diploid tumours ($n$ = 129) subdivided according to number of AgNORs

| *Category* | *Number* | *RFP (s.e.)* | *P-value*[a] |
|---|---|---|---|
| *All cases* | | | |
| AgNOR≤3.5 | 160 | 4.2 (0.3) | |
| AgNOR>3.5 | 69 | 2.5 (0.4) | 0.0010 |
| *Papillary tumours* | | | |
| AgNOR≤3.5 | 146 | 4.4 (0.4) | |
| AgNOR>3.5 | 44 | 2.8 (0.5) | 0.0125 |
| *Diploid tumours* | | | |
| AgNOR≤3.5 | 103 | 4.4 (0.4) | |
| AgNOR>3.5 | 26 | 2.3 (0.6) | 0.0045 |
| *SPF* | | | |
| SPF≤10.0% | 127 | 4.1 (0.4) | |
| SPF>10.0% | 66 | 2.3 (0.4) | 0.0010 |

[a]Student's *t*-test. For comparison the RFP subdivided according to SPF is shown.

**Table VI** The mean (s.e.) number of AgNORs in tumours leading to bladder cancer death, in patients dying of intercurrent diseases and in patients alive at the end of the follow-up

| *Subgroup* | *Number* | *AgNORs (s.e.)* | *P-value*[a] |
|---|---|---|---|
| *All cases* | | | |
| Bladder cancer | 73 | 3.7 (0.2) | |
| Other diseases | 72 | 2.9 (0.2) | <0.0001 |
| Alive | 84 | 2.7 (0.1) | |
| *Papillary tumours* | | | |
| Bladder cancer | 45 | 3.3 (0.2) | |
| Other diseases | 67 | 2.7 (0.1) | 0.0038 |
| Alive | 78 | 2.6 (0.1) | |
| *Ta-T1 tumours* | | | |
| Bladder cancer | 14 | 3.4 (0.5) | |
| Other diseases | 47 | 2.9 (0.2) | 0.0226 |
| Alive | 59 | 2.4 (0.1) | |

[a]Two-tailed analysis of variance.

**Figure 2** Disease related survival of all patients subdivided according to number of AgNORs. The difference in survival between curves is significant ($\chi^2$ = 12.1, $P$ = 0.0005). Curve A: $n$ = 160, AgNOR number≤3.5; Curve B: $n$ = 69, AgNOR number>3.5.

(Table IV). AgNORs were related to RFP (Table V) whereas the RR could not be predicted by AgNORs. WHO grade ($P$ = 0.0582), DNA ploidy ($P$ = 0.0261) and SPF ($P$ = 0.0747) were related to RFP. AgNORs predicted disease related survival significantly (Table VI, Figure 4). In multivariate analysis AgNORs had no independent predictive value.

### *Ta-T1 and T2-T3 tumours*

A significant relationship between AgNOR count, papillarity ($P$ = 0.0060), WHO grading ($P$<0.0001), DNA ploidy ($P$ = 0.0700) and SPF ($P$ = 0.0028) was present. Progression in T- ($P$ = 0.12), N ($P$ = 0.16) and M- categories ($P$ = 0.11) was related to AgNORs with a borderline significance. In a logistic multiparameter analysis AgNORs had independent prognostic values as predictors of progression (Table IV). the RR ($P$ = 0.12) and RFP ($P$ = 0.18) were related to AgNORs with a borderline significance. WHO grade ($P$ = 0.118), DNA ploidy ($P$ = 0.069), SPF ($P$ = 0.065) and papillarity ($P$ = 0.098) predicted RFP. DNA ploidy ($P$ = 0.0370) and SPF ($P$ = 0.0500) were significant predictors of RR. In diploid tumours the RR was 2.5 (0.4) *vs* 5.1 (1.1) of aneuploidy tumours ($n$ = 26). Tumours with SPF≤15.0% had a mean RR of 2.6 (0.4) in comparison to 10.4 (3.0) in tumours with SPF>15.0%. In diploid tumours AgNORs predicted RFP to some degree ($P$ = 0.20). In univariate survival analysis

**Figure 3** Disease related survival of patients with a diploid bladder cancer subdivided according to number of AgNORs. The difference in survival between the curves is significant ($\chi^2 = 14.5$, $P = 0.0001$). Curve A: $n = 103$, AgNOR number $\leqslant 3.5$; Curve B: $n = 26$, AgNOR number $> 3.5$.

**Figure 5** Disease related survival of patients with superficial bladder cancer subdivided according to number of AgNORs. The difference in survival between the curves is almost significant ($\chi^2 = 3.2$, $P = 0.0751$). Curve A: $n = 97$, AgNOR number $\leqslant 3.5$; Curve B: $n = 23$, AgNOR number $> 3.5$.

**Figure 4** Disease related survival of patients with papillary bladder cancer subdivided according to number of AgNORs. The difference in survival between the curves is significant ($\chi^2 = 8.4$, $P = 0.0038$). Curve A: $n = 146$, AgNOR number $\leqslant 3.5$; Curve B: $n = 44$, AgNOR number $> 3.5$.

AgNORs predicted disease related survival (Figure 5) whereas in multivariate analysis they had no independent predictive value.

In T2-T3 tumours AgNORs predicted progression in univariate analysis ($P = 0.0410$) whereas they had no independent predictive value in multivariate analysis. Non-progressing tumours (T) ($n = 49$) had 3.0 (0.2) AgNORs whereas progressing tumours ($n = 43$) had a mean of 3.7 (0.2) AgNORs. AgNORs predicted bladder cancer related survival in univariate analysis ($\chi^2 = 11.1$, $P = 0.004$). In a multivariate analysis they had no independent predictive value.

### Discussion

The present series has previously been analysed by morphometry (Lipponen & Eskelinen, 1990*a*, 1990*b*; Lipponen *et al.*, 1990*c*) and DNA flow cytometry (Lipponen *et al.*, 1991*a*). The predictive value of clinical stage, papillarity and WHO grade has been described in connection with these reports. So, special emphasis is given to AgNORs which are subject to controversies as prognostic variables or indicators of proliferative activity in several malignancies (Giri *et al.*, 1989; Rushoff *et al.*, 1990*a*; Sivridis & Sims, 1990; Delahut *et al.*, 1991; Eskelinen *et al.*, 1991*b*) including transitional cell bladder cancer (Cairns *et al.*, 1989; Ooms & Veldhuizen, 1989; Mansour *et al.*, 1990; Lipponen & Eskelinen, 1991*a*).

The AgNORs are located in acrocentric chromosomes, each chromosome having two AgNORs. All AgNORs are not visible in normal histological sections and usually one or two may be present within the nucleus (Underwood & Giri, 1988). Accordingly 20 AgNORs may be visible in normal nucleus before mitosis. Since in aneuploid cells the number of chromosomes at any phase of cell cycle is higher than in diploid cells higher AgNOR counts can be found in case additional chromosomal material bears NOR sites. However, AgNORs present active rRNA (Wachlter *et al.*, 1986) and the proliferative activity of a given cell determines the number of AgNORs suggesting a relationship between the number of AgNORs and SPF.

Aneuploid tumours as well as tumours with high SPF had significantly higher numbers of AgNORs than diploid tumours with low SPF. The results are in agreement with the results in breast tumours (Giri *et al.*, 1989; Eskelinen *et al.*, 1991*b*). Consequently, high grade tumours, non-papillary tumours and muscle invasive tumours had higher AgNOR counts since most of these tumours are aneuploid (Lipponen *et al.*, 1991*b*). The relationship between grade, papillarity and AgNOR counts has been presented previously (Cairn *et al.*, 1989; Ooms & Veldhuizen, 1989; Lipponen & Eskelinen, 1991*a*) the present results supporting these findings. The relationship between DNA flow cytometric data and AgNORs has not been reported previously in transitional cell bladder tumours.

The AgNORs were able to predict pelvic lymph node involvement at the time at diagnosis as well as they predict axillary lymph node involvement in breast cancer (Sivridis & Sims, 1990). The potential of AgNORs to predict pretreatment lymph node metastasis is similar to that of DNA ploidy and SPF in the same clinical material (Lipponen *et al.*, 1991*b*). Moreover, AgNORs were related significantly to progression postoperatively the results being in line with those obtained by mitotic indexes (Lipponen & Eskelinen, 1990*b*, Lipponen *et al.*, 1990*c*) and DNA flow cytometry (Lipponen *et al.*, 1991*b*). The potential to predict progression in Ta-T1 tumours may permit a more precise stratification of these tumours like mitotic indexes (Lipponen *et al.*, 1990*c*) or flow cytometric data (Lipponen *et al.*, 1991*b*). WHO grading seems to be of rather limited value in predicting progression in individual cases (Lipponen *et al.*, 1990*c*). This is supported by the ability of AgNORs to regroup WHO grade III tumours in terms of progression. These latter results are contradictory to observations presented previously (Mansour *et al.*, 1990), however, their series included 11 patients with a short follow-up.

Aneuploid bladder tumours with high SPF reccur more often having usually a shorter RFP than diploid ones (Blomjous *et al.*, 1989) whereas WHO grade is a weak predictor of RFP (Lipponen & Eskelinen, 1990*a*). In the present analysis AgNORs predicted REF like SPF, DNA index or mitotic activity (Lipponen *et al.*, 1990*c*). It was unexpected that RFP of diploid tumours could be further regrouped according to their AgNOR number. This latter finding may be related to intratumour heterogenity of DNA ploidy (Lipponen *et al.*, 1991*b*) or AgNORs are independent of DNA ploidy since the proliferative status of a given cell determines the AgNOR number (Wachtler *et al.*, 1986). Aneuploid tumours could not be regrouped, however. The relationship between AgNORs and RFP in bladder cancer is consonant to results in breast cancer in which AgNORs predict significantly RFP, too (Eskelinen *et al.*, 1991*b*). The differences in RR and RFP could not be attributed to intravesical chemotherapy since it had not significant predictive value in univariate analysis ($P = 0.213$) or in multivariate analysis.

AgNORs were related to progression consequently predicting disease related survival. In colon tumours (Rushoff *et al.*, 1990*a*; Öfner *et al.*, 1990) and in renal cell tumours (Delahut *et al.*, 1991). AgNORs have been able to predict survival significantly even within clinical stage categories (Delahut *et al.*, 1991). Accordingly, AgNORs predicted survival in papillary bladder tumours, in superficial tumours and in muscle invasive tumours. Surprisingly, diploid bladder cancers could be stratified. As with recurrences, this latter result is with a higher probability related to intratumour heterogenety of DNA ploidy (Lipponen *et al.*, 1991*b*). On the other hand, SPF can regroup diploid tumours which suggest subgroups of diploid bladder tumours with different proliferative potentials. Patients dying of their bladder cancer had higher AgNOR counts than patients dying of intercurrent diseases or being alive after follow-up. However, in a multivariate analysis of survival including clinical, histological, flow cytometric variables and AgNORs, the AgNORs had no independent prognostic value.

The methodology in the present analysis differs from that of many other analyses presented until now (Cairns *et al.*, 1989; Mansour *et al.*, 1990; Lipponen & Eskelinen, 1991*a*). Firstly, the areas of analysis were selected aiming at finding the most atypical fields for measurement whereas previous studies have used random sampling. We feel that selective sampling led us to improved predictive results. The importance of selection of fields for analysis cannot be overemphasised since bladder tumours often show intratumour heterogenity of malignancy (Lipponen *et al.*, 1991*b*). In accordance with the above selective morphometry (Lipponen & Eskelinen, 1990*a*, Lipponen & Eskelinen, 1990*b*) has given good prognostic results in bladder cancer. Secondly, 5 μm thick sections were used in contrast to 3 μm thick sections were used in contrast to 3 μm sections used in most studies. In 3 μm sections dispersed AgNORs free within the nucleus may be lost and the data are 'compressed' between high- and low-count specimens (Crocker *et al.*, 1989). In the present analysis the microscope was focused to find the maximum number of AgNORs visible at the same time. The number of nuclei counted was limited to 70 since the counting of AgNORs is time consuming and moreover the methodological studies have shown that the standard error of the mean does not vary significantly after 50–60 nuclei has been counted (Rushoff *et al.*, 1990*b*). In the present analysis the intraobserver variation of the mean was $5 \times \%$ which is comparable to results observed by other researchers (Mansour *et al.*, 1990; Rushoff *et al.*, 1990*b*; Sivridis & Sims, 1990).

From the results we can conclude that AgNORs are related to proliferative activity and malignancy in bladder cancer. AgNORs have independent prognostic value in superficial bladder tumours as predictors of progression. In muscle invasive tumours classic prognostic factors and flow cytometric variables are more important predictors. The results suggest that AgNORs can be used as an adjunct to histological grading, flow cytometry and mitotic indexes in predicting clinical behaviour in superficial bladder tumours. It is hard to imagine the use of AgNORs alone in predicting individual cases of bladder cancer due to considerable overlap in AgNOR counts between malignant and more benign bladder tumours. The results encourage for further studies giving special emphasis to standardisation of measurement process and this refers to morphometric methods, in particular (Rushoff *et al.*, 1990*a*; Rushoff *et al.*, 1990*b*).

The study was supported by a research grant from Savon Syöpärahasto. The assistance of Mrs A.-L. Gidlund is gratefully acknowledged.

### References

BLOMJOUS, E.C.M., SCHIPPER, N.W., BAAK, J.P.A., VOS, W., DE VOOGT, H.J. & MEIJER, C.J.L.M. (1989). The value of morphometry and DNA flow cytometry in addition to classic prognosticators in superficial urinary bladder carcinomas. *Am. J. Clin. Pathol.*, **91,** 243.

CAIRNS, P., SUAREZ, V., NEWMAN, J. & CROCKER, J. (1989). Nucleolar organiser regions in transitional cell tumors of the bladder. *Arch. Pathol. Lab. Med.*, **113,** 1250.

CROCKER, J., BOLDY, D.A.R. & EGAN, M.J. (1989). How should we count AgNORs? Proposals for a standardized approach. *J. Pathol.*, **158,** 185.

DELAHUT, B., RIBAS, J.L., NACEY, N.J. & BATHWAITE, P.B. (1991). Nucleolar organiser regions and prognosis in renal cell carcinoma. *J. Pathol.*, **163,** 31.

ESKELINEN, M.J., LIPPONEN, P.K., COLLAN, Y., MARIN, S., ALHAVA, E. & NORDLING, S. (1991*a*). Relationship between DNA ploidy and survival in patients with exocrine pancreatic cancer. *Pancreas*, **6,** 90.

ESKELINEN, M.J., LIPPONEN, P.K., COLLAN, Y. & SYRJÄNEN, K. (1991*b*). The role of nucleolar organiser regions (Ag-NORs) as prognostic factors in breast cancer. *Eur. J. Cancer*, **27,** 989.

GIRI, D.D., NOTTINGHAM, J.F., LAWRY, J., DUNDAS, S.A.C. & UNDERWOOD, J.C.E. (1989). Silver-binding nucleolar organizer regions (AgNORs) in benign and malignant breast lesions: correlations with ploidy and growth phase by DNA flow cytometry. *J. Pathol.*, **157,** 307.

LEE, E. & DESU, M. (1972). A computer program for comparing k samples with right censored data. *Com. Prog. Biomed.*, **2,** 315.

LIPPONEN, P.K. & ESKELINEN, M.J. (1990*a*). Nuclear morphometry in grading transitional cell bladder cancer compared with subjective histological grading. *Anticancer Res.*, **10**, 1725.

LIPPONEN, P.K. & ESKELINEN, M.J. (1990*b*). Volume-corrected mitotic index and mitotic activity index in transitional cell bladder cancer. *Eur. Urol.*, **18**, 258.

LIPPONEN, P.K. & ESKELINEN, M.J. (1991*a*). Nucleolar organiser regions (NORs) in bladder cancer; relation to histological grade, clinical stage and prognosis. *Anticancer Res.*, **11**, 75.

LIPPONEN, P.K., ESKELINEN, M.J. & SOTARAUTA, M. (1990*c*). Prediction of superficial bladder cancer by histoquantitative methods. *Eur. J. Cancer*, **26**, 1060.

LIPPONEN, P.K., ESKELINEN, M.J. & NORDLING, S. (1991*b*). Progression and survival in transitional cell bladder cancer. A comparison of established prognostic factors, S-phase fraction and DNA ploidy. *Eur. J. Cancer*, **27**, 877.

LIPPONEN, P.K., ESKELINEN, M.J. & NORDLING, S. (1991*c*). The relationship between DNA flow cytometric data, nuclear morphometric variables and volume corrected mitotic index (M/V index) in transitional cell bladder tumours. *Eur. Urol.*, **19**, 327.

MANSOUR, P., CROCKER, J. & NEWMAN, J. (1990). Lack of prognostic value of nucleolar organiser region enumeration in transitional cell bladder carcinoma of the bladder. *Arch. Pathol. Lab. Med.*, **114**, 1261.

MOSTOFI, F.K. (1973). International histological classification of tumours. In *Histological Typing of Urinary Bladder Tumours*, No. 10, WHO, Geneva.

ÖFNER, D., TÖTSCH, M., SANDBICHLER, P. & 4 others (1990). Silver stained nucleolar organiser region proteins (Ag-NORs) as a predictor of prognosis in colonic cancer. *J. Pathol.*, **162**, 43.

OOMS, E.C.M., ANDERSSON, W.A.D., ALONS, C.L., VELDHUIZEB, R.W. & BOON, M.E. (1983). An analysis of the performance of pathologists in grading bladder tumors. *Hum. Pathol.*, **14**, 140.

OOMS, E.C.M. & VELDHUIZEN, R.W. (1989). Argyrophilic proteins of the nucleolar organizer region in bladder tumours. *Virchows Archiv [A] Pathol. Anat.*, **414**, 365.

RUSHOFF, J., BITTINGER, A., NEUMANN, K. & SCHMITZ-MOORMAN, P. (1990*a*). Prognostic significance of nucleolar organizing regions (NORs) in carcinomas of the sigmoid colon and rectum. *Path. Res. Pract.*, **186**, 85.

RUSHOFF, J., PLATE, K.H., CONTRACTOR, H., KERN, S., ZIMMERMAN, R. & THOMAS, K. (1990*b*). Evaluation of nucleolus organizer regions (NORs) by automatic image analysis: a contribution to standardization. *J. Pathol.*, **161**, 113.

SIVRIDIS, E. & SIMS, B. (1990). Nucleolar organiser regions: new prognostic variable in breast carcinomas. *J. Clin. Pathol.*, **3**, 390.

SMITH, R. & CROCKER, J. (1988). Evaluation of nucleolar organiser region associated proteins in breast malignancy. *Histopathology*, **12**, 113.

UICC (UNION INTERNATIONALE CONTRE LE CANCER) (1978). TNM classification of malignant tumours. Third edition. International Union Against Cancer, Geneva.

UNDERWOOD, J.C.E. & GIRI, D.D. (1988). Nucleolar organiser regions as diagnostic discriminants for malignancy. *J. Pathol.*, **155**, 95.

WACHTLER, F., HOPMAN, A.H.N., WIGAT, J. & SCHWARTZACHER, H.G. (1986). On the position of nuleolus organizer regions (NORs) in interphase nuclei. *Exp. Cell. Res.*, **167**, 227.

ZINGG, E.J. & WALLACE, D.M.A. (1985). (eds) Clinical practice in urology. *Bladder Cancer*, pp. 161–191. Springer-Verlag: Heidelberg.